# What do colorectal specialists think about female participation in anal intercourse? An online survey of UK coloproctologists

**DOI:** 10.1007/s10151-025-03202-7

**Published:** 2025-08-16

**Authors:** Tabitha Gana, Lesley Hunt

**Affiliations:** 1 Lancaster University, Lancaster, LNH LA1 4YW UK; 2 Bradford Teaching Hospitals NHS Foundation Trust, Duckworth Lane, Bradford, BD9 6RJ UK; 3 Sheffield Teaching Hospitals NHS Foundation Trust, Herries Road, Sheffield, S5 7AU UK

## Abstract

**Background:**

Increasing participation in anal intercourse (AI) raises questions about its effects on the female anus. Societal change has moved faster than published literature.

**Method:**

Online survey of Association of Coloproctology of Great Britian & Ireland (ACPGBI) and Association of Surgeons of Great Britain & Ireland (ASGBI) members to document clinical practice regarding female AI; opinion on female AI in causation of anal pathology; barriers to discussing AI; possible harms and harm reduction and public information.

**Results:**

91% of consultant colorectal surgeons (CCS) agree female AI causes anal fissures. Only 25% usually or always ask young women with fissures about AI and 31% never ask. Enquiry increases with refractory fissures (34%) and vulnerable patients (57%); 48% of CCS cite fear of patient discomfort, and 40% fear what the patient thinks of them as barriers to enquiry.

Eighty per cent of CCS and 85% of pelvic floor specialists (PFS) agree AI can cause internal anal sphincter (IAS) damage and 72% and 78% faecal incontinence (FI) in women. Eleven per cent of CCS and no PFS agreed relaxation techniques, and 17% and 14% lubrication, protect the IAS; 97% of CCS think there should be increased public health awareness about female AI.

**Conclusions:**

Experts think participation in AI can cause fissures, IAS damage and FI in women. They are sceptical about the protective value of lubrication and relaxation. Clinical conversations lag behind experts’ opinions on the importance and possible consequences of female AI. Concern over patients’ feelings are barriers to enquiry. Colorectal specialists think there should be more public health information about female AI.

## Introduction

Over the past 20 years, female participation in anal intercourse (AI) had increased to 44% of 20–44-year-old US women in 2016 [1–3]. The UK National Surveys of Sexual Attitudes and Lifestyles (NatSALs) found participation rose from 12.5% in 1990 [4] to 28.5% in 2012 [5]. The 2022 survey did not report AI [4]; however, media coverage suggests participation has increased further. Portrayal of female AI is widespread across pornography, popular culture and the media, both reflecting and influencing attitudes [6].

Medical research and publications have not kept pace with societal change, presenting difficulties for clinicians managing women with anal conditions. Public health education is lacking [7, 8]. NHS patient information solely discusses sexually transmitted diseases [9].

Although female AI is often consensual, many of the reasons women cite for engaging do not signify agency. Motivation is often focussed on male pleasure, relationship maintenance and meeting societal norms [10–13]. Those encouraging and even coercing women to participate may not be aware of the potential effects.

Our primary objective was to explore the opinions and practice of those caring for women with anal pathology regarding female anal penetration. Where definitive evidence is not yet available, expert opinion becomes best available evidence. The opinions of specialists can guide patients, other clinicians, the public and health educators. This is particularly important in female AI as there is a plethora of unsubstantiated information and pseudo-medical advice available elsewhere.

## Aims

To document clinical history taking by those caring for women with anal pathology potentially associated with AI and the importance attached by colorectal specialists to specific enquiry about AI.

To ascertain barriers to patient conversations.

To ascertain the opinions of clinicians about the significance of AI in the aetiology of anal pathology, potential harms of female AI, the value of suggested methods for harm reduction, clinician training and public health information.

## Method

This survey was conducted and the results presented in accordance with the Consensus-based Checklist for Reporting of Survey Studies (CROSS) [14]. Data were obtained by a single online questionnaire. After pilot testing, participant information, consent and a questionnaire (Appendix 1) were distributed to a non-purposive sample of healthcare practitioners managing outpatients with anorectal disease, via the Association of Coloproctology and Association of Surgeons GB and Ireland websites, social media and mailing lists, using Qualtrics secure web application (Qualtrics © 2023, Provo, UT, USA). Completion time was 5 min. Replies between September 2023 and February 2024 were analysed. Participants could complete the survey only once.

All respondents saw the same stand-alone and matrix table closed end multiple choice questions on:

1. Demographics and diversity.
2. Role and practice.
3. Opinions about female AI: (a) history taking, (b) clinical practice, (c) ease talking about sexual matters and (d) barriers to female AI enquiry.
4. Possible adverse effects and protection.
5. Training and public health information.

Respondents estimated UK prevalence of female participation in AI (linear analogue scale 1–100%).

No qualitative questions were used.

Pre-defined survey population: consultants, trust doctors, surgical trainees, advanced care practitioners and nurse specialists seeing < 1 patient with anorectal disease per week. Roles and subspeciality were recorded. Responses were analysed with focus on the opinions of consultant colorectal surgeons (CCS) and pelvic floor surgeons (PFS).

Questionnaires were submitted anonymously and identified by study number. IP addresses were not collected. Data were held securely on password-protected systems. Reasons for non-participation were not collected. Data were collated and analysed in Excel © version 16.86 with statistical analysis in SPSS © version 29.

## Results

There were 435 unique survey site visits; 178 individuals completed; 11 respondents saw < 1 patient a week with an anal condition or rectal bleeding and were excluded from analysis; 167 were analysed (69 female, 98 male); 87 (52%) saw 1–10; 41 (25%) 10–20 and 27 (16%) > 20 patients with anal conditions or rectal bleeding a week. Age, ethnicity, religion and sexual orientation are given in Table 1. Speciality was described using ≤ 1 closed responses: 114 (68%) Colorectal or Colorectal + other or + Pelvic Floor Specialist (PFS); 53 (32%) General or Emergency Surgeons or non-surgical; 75 current Consultant Colorectal Surgeons (CCS), 14 specifying PFS. Most respondents accurately estimated the prevalence of the AI rate for women aged 34 [median 35% (range 5–88%)] years regarding all respondents; 33% (range 10–81%) CCS; 40% (range 17–71%) PFS.

**Table 1:** Age, ethnicity, religion, sexual orientation and role of respondents

Respondents age, ethnicity, religion, sexual orientation and role	
Age group (years)	Number (%)
21–30	12 (7)
31–40	62 (37)
41–50	42 (25)
51–60	38 (23)
> 60	13 (8)
Ethnicity	Number (%)
Asian, British Asian	35 (21)
Black, Black British, Caribbean, African	12 (7)
Other, Mixed	10 (10)
White	110 (66)
Religious belief	Number (%)
Christian	55 (32)
Hindu	11 (7)
Muslim	21 (13)
None	73 (44)
Other, prefer not to say	7 (4)
Sexual orientation	Number (%)
Bisexual	8 (5)
Heterosexual	149 (89)
Homosexual	4 (2)
Other	3 (2)
Prefer not to say	3 (2)
Current role	Number (%)
Colorectal nurse specialist/advanced nurse practitioner	15 (9)
Consultant surgeon	90 (54)
Surgical trainee or staff in first 2 years of training	13 (8)
Higher surgical trainee or equivalent in years 3–5 of training	24 (14)
Higher surgical trainee or equivalent in years 6–8 of specialist training	18 (11)
Non-consultant grade surgeon with > 8 years’ experience	7 (4)

Opinions regarding the possible adverse effects of female AI are shown in Table 2. Opinions regarding the importance of enquiry about AI in women presenting with anal symptoms are shown in Table 3, and respondents’ current clinical practice of enquiry is shown in Table 4. There was agreement about possible pathological effects of female AI but less about the importance of discussion. Discrepancy was seen between stated importance of asking and actually asking.

**Table 2:** Opinion regarding possible adverse effects of female AI

To what extent do you agree with the following statements?”						
	All respondents (%) *n* = 167		Colorectal consultants (%) *n* = 75		Pelvic floor specialists (%) *n* = 14	
Anal sex can lead to mucosal tears/fissures in women	Completely agree	100 (60)	Completely agree	47 (63)	Completely agree	8 (57)
	Agree	39 (23)	Agree	21 (28)	Agree	4 (28)
	Neither agree or disagree	20 (12)	Neither agree or disagree	3 (4)	Neither agree or disagree	1 (7)
	Disagree	1 (1)	Disagree	1 (1)	Disagree	0
	Completely disagree	1 (1)	Completely disagree	0	Completely disagree	0
	Don’t know	6 (4)	Don’t know	1 (1)	Don’t know	1 (7)
Anal sex can lead to anal pain in women	Completely agree	96 (57)	Completely agree	48 (64)	Completely agree	10 (71)
	Agree	45 (27)	Agree	19 (25)	Agree	3 (21)
	Neither agree or disagree	13 (8)	Neither agree or disagree	3 (4)	Neither agree or disagree	0
	Disagree	3 (2)	Disagree	1 (1)	Disagree	0
	Completely disagree	1 (1)	Completely disagree	0	Completely disagree	0
	Don’t know	9 (5)	Don’t’ know	4 (5)	Don’t know	1(7)
Anal sex can lead to anal bleeding in women	Completely agree	99 (59)	Completely agree	45 (60)	Completely agree	9 (64)
	Agree	42 (25)	Agree	22 (29)	Agree	2 (14)
	Neither agree or disagree	14 (8)	Neither agree or disagree	5 (7)	Neither agree or disagree	1 (7)
	Disagree	5 (3)	Disagree	1 (1)	Disagree	0
	Completely disagree	2 (1)	Completely disagree	1 (1)	Completely disagree	1 (7)
	Don’t know	5 (3)	Don’t know	1 (1)	Don’t know	0
Anal sex can lead to damage of the internal anal sphincter in women	Completely agree	78 (47)	Completely agree	43 (57)	Completely agree	8 (57)
	Agree	42 (25)	Agree	17 (23)	Agree	4 (28)
	Neither agree or disagree	23 (14)	Neither agree or disagree	7 (9)	Neither agree or disagree	1 (7)
	Disagree	7 (4)	Disagree	2 (3)	Disagree	0
	Completely disagree	2 (1)	Completely disagree	1 (1)	Completely disagree	0
	Don’t know	15 (9)	Don’t know	5 (7)	Don’t know	1 (7)
Anal sex can lead to incontinence in women	Completely agree	70 (42)	Completely agree	38 (51)	Completely agree	7 (50)
	Agree	39 (23)	Agree	16 (21)	Agree	4 (28)
	Neither agree or disagree	33 (20)	Neither agree or disagree	12 (16)	Neither agree or disagree	2 (14)
	Disagree	2 (1)	Disagree	1 (1)	Disagree	0
	Completely disagree	17 (10)	Completely disagree	1 (1)	Completely disagree	0
	Don’t know	39 (23)	Don’t know	7 (9)	Don’t know	1 (7)
Women are at greater risk of incontinence after having anal sex c.f. men	Completely agree	33 (20)	Completely agree	21 (28)	Completely agree	5 (36)
	Agree	28 (17)	Agree	14 (19)	Agree	3 (21)
	Neither agree or disagree	26 (16)	Neither agree or disagree	7 (9)	Neither agree or disagree	1 (7)
	Disagree	18 (11)	Disagree	10 (13)	Disagree	2 (14)
	Completely disagree	7 (14)	Completely disagree	3 (4)	Completely disagree	0
	Don’t know	55 (33)	Don’t know	20 (27)	Don’t know	2 (14)

**Table 3:** Opinions regarding the importance of enquiry about AI in women presenting with anal symptoms

“To what degree do you agree with the following statements?”						
	All respondents *n* = 167 (%)		Colorectal consultants (%) *n* = 75		Pelvic floor specialists (%) *n* = 14	
Asking about anal sex is important in all women with anal symptoms	Strongly agree	28 (17)	Strongly agree	9 (12)	Strongly agree	3 (21)
	Agree somewhat	26 (16)	Agree somewhat	15 (20)	Agree somewhat	3 (21)
	Neither agree or disagree	56 (34)	Neither agree or disagree	28 (37)	Neither agree or disagree	5 (36)
	Disagree somewhat	25 (15)	Disagree somewhat	12 (16)	Disagree somewhat	1 (7)
	Strongly disagree	10 (6)	Strongly disagree	4 (5)	Strongly disagree	0
	Not sure	22 (13)	Not sure	7 (9)	Not sure	2 (14)
Asking about anal sex is important in women under 40 years with anal symptoms	Strongly agree	34 (20)	Strongly agree	17 (23)	Strongly agree	5 (36)
	Agree somewhat	37 (22	Agree somewhat	16 (21)	Agree somewhat	4 (29)
	Neither agree or disagree	51 (31)	Neither agree or disagree	26 (35)	Neither agree or disagree	3 (21)
	Disagree somewhat	17 (10)	Disagree somewhat	7 (9)	Disagree somewhat	0
	Strongly disagree	7 (4)	Strongly disagree	2 (3)	Strongly disagree	0
	Not sure	21 (13)	Not sure	7 (9)	Not sure	2 (14)
Asking about anal sex is important in all women with faecal incontinence	Strongly agree	42 (25)	Strongly agree	17 (23)	Strongly agree	5 (36)
	Agree somewhat	26 (16)	Agree somewhat	10 (13)	Agree somewhat	3 (21)
	Neither agree or disagree	45 (27)	Neither agree or disagree	23 (31)	Neither agree or disagree	3 (21)
	Disagree somewhat	25 (15) 10 (6)	Disagree somewhat	12 (16)	Disagree somewhat	1 (7)
	Strongly disagree	19 (11)	Strongly disagree	6 (8)	Strongly disagree	0
	Not sure		Not sure	7 (9)	Not sure	2 (14)
Asking about anal sex is important in women under 40 years with faecal incontinence	Strongly agree	54 (32)	Strongly agree	14 (19)	Strongly agree	7 (50)
	Agree somewhat	37 (22)	Agree somewhat	25 (33)	Agree somewhat	3 (21)
	Neither agree or disagree	34 (20)	Neither agree or disagree	20 (27)	Neither agree or disagree	2 (14)
	Disagree somewhat	18 (11)	Disagree somewhat	6 (8)	Disagree somewhat	0
	Strongly disagree	6 (4)	Strongly disagree	2 (3)	Strongly disagree	0
	Not sure	18 (11)	Not sure	8 (11)	Not sure	2 (14)
Asking about anal sex is important in nulliparous women with faecal incontinence	Strongly agree	59 (35)	Strongly agree	27 (36)	Strongly agree	7 (50)
	Agree somewhat	34 (20)	Agree somewhat	15 (20)	Agree somewhat	2 (14)
	Neither agree or disagree	30 (18)	Neither agree or disagree	19 (25)	Neither agree or disagree	3 (21)
	Disagree somewhat	12 (7)	Disagree somewhat	6 (8)	Disagree somewhat	0
	Strongly disagree	9 (5)	Strongly disagree	2 (3)	Strongly disagree	0
	Not sure	23 (14)	Not sure	6 (8)	Not sure	2 (14)

**Table 4:** Respondents’ current clinical practice of enquiry about AI

“How often would you enquire about anal sex in the following scenarios?”						
	All respondents (%) *n* = 167		Colorectal consultants (%) *n* = 75		Pelvic floor specialists (%) *n* = 14	
19-year-old woman presenting with typical anal fissure?	Always	12 (7)	Always	4 (5)	Always	1 (7)
	Most of the time	24 (14)	Most of the time	15 (20)	Most of the time	4 (29)
	About half of the time	11 (7)	About half of the time	5 (7)	About half of the time	3 (21)
	Sometimes	51 (31)	Sometimes	28 (37)	Sometimes	3 (21)
	Never	68 (41)	Never	23 (31)	Never	3 (21)
	Never encounter this scenario	1 (1)	Never encounter this scenario	0	Never encounter this scenario	0
30-year-old woman with a refractory anal fissure?	Always	23 (14)	Always	13 (17)	Always	5 (36)
	Most of the time	23 (14)	Most of the time	13 (17)	Most of the time	2 (14)
	About half of the time	15 (9)	About half of the time	8 (11)	About half of the time	2 (14)
	Sometimes	56 (34)	Sometimes	27 (36)	Sometimes	3 (21)
	Never	48 (29)	Never	14 (19)	Never	2 (14)
	Never encounter this scenario	2 (1)	Never encounter this scenario	0	Never encounter this scenario	0
Nulliparous 25-year-old with passive leak incontinence?	Always	24 (14)	Always	15 (20)	Always	6 (43)
	Most of the time	30 (18)	Most of the time	16 (21)	Most of the time	2 (14)
	About half of the time	16 (10)	About half of the time	10 (13)	About half of the time	2 (14)
	Sometimes	49 (29)	Sometimes	23 (31)	Sometimes	2 (14)
	Never	42 (25)	Never	11 (15)	Never	2 (14)
	Never encounter this scenario	6 (4)	Never encounter this scenario	0	Never encounter this scenario	0
Multiparous 40-year-old with passive leak incontinence	Always	14 (8)	Always	6 (8)	Always	3 (21)
	Most of the time	11 (7)	Most of the time	7 (9)	Most of the time	0
	About half of the time	14 (8)	About half of the time	7(9)	About half of the time	2 (14)
	Sometimes	61 (37)	Sometimes	36 (48)	Sometimes	6 (43)
	Never	64 (38) 3(2)	Never	19 (25)	Never	3 (21)
	Never encounter this scenario		Never encounter this scenario	0	Never encounter this scenario	0
22-year-old woman considering a pouch for ulcerative colitis	Always	30 (18)	Always	19 (25)	Always	3 (21)
	Most of the time	12 (7)	Most of the time	4 (5)	Most of the time	2 (14)
	About half of the time	4 (2)	About half of the time	1 (1)	About half of the time	0
	Sometimes	24 (14)	Sometimes	15 (20)	Sometimes	1 (7)
	Never	57 (34)	Never	11 (15)	Never	1 (7)
	Never encounter this scenario	40 (24)	Never encounter this scenario	25 (33)	Never encounter this scenario	7 (50)
Vulnerable 25-year-old woman presenting with anal pain	Always	48 (29)	Always	24 (32)	Always	8 (57)
	Most of the time	36 (26)	Most of the time	19 (25)	Most of the time	0
	About half of the time	13 (8)	About half of the time	10 (13)	About half of the time	2 (14)
	Sometimes	37 (22)	Sometimes	14 (19)	Sometimes	2 (14)
	Never	28 (17)	Never	8 (11)	Never	2 (14)
	Never encounter this scenario	5 (3)	Never encounter this scenario	0	Never encounter this scenario	0

As evidenced by being more likely to respond always or most of the time (labelled usually) to the following scenarios—a 19-year-old woman presenting with typical anal fissure, a 30-year-old woman with a refractory anal fissure and a nulliparous 25-year-old with passive leak incontinence—there was no association among clinician age, ethnicity or religious affiliation and practice of asking about AI. [Some categories required grouping of characteristics (white vs non-white and any religious affiliation vs non-religious.)] Clinician sex influenced enquiry; 30% of female clinicians c.f. 15% male clinicians usually ask a 19-year-old woman presenting with typical anal fissure (*p* = 0.021) and 43% versus 27% a nulliparous 25-year-old with passive leak incontinence (*p* = 0.027). For a 30-year-old woman with a refractory anal fissure, enquiry by clinicians of both sexes increased (35% female vs 23% male) and there was no difference between male and female clinicians (*p* = 0.075).

Younger surgeons are more likely to be female, ethnically and culturally diverse. Logistic regression analysis showed no correlation between clinician age, ethnicity or religious affiliation and the practice of asking about AI. Clinician sex remained a significant predictor of practice. Female clinicians are 2.1 times more likely than male colleagues to ask a nulliparous 25-year-old woman with a passive leak incontinence about AI (95% CI 1.08 to 4.01, *p* = 0.028) and 2.4 times more likely to ask a 19-year-old woman presenting with typical anal fissures about it (95% CI 1.13 to 5.08, *p* = 0.023). There was no difference between male and female clinicians' enquiry rates for a 30-year-old woman presenting with refractory anal fissures (95% CI 0.269 to 1069, *p* = 0.077).

**Fig. 1:**
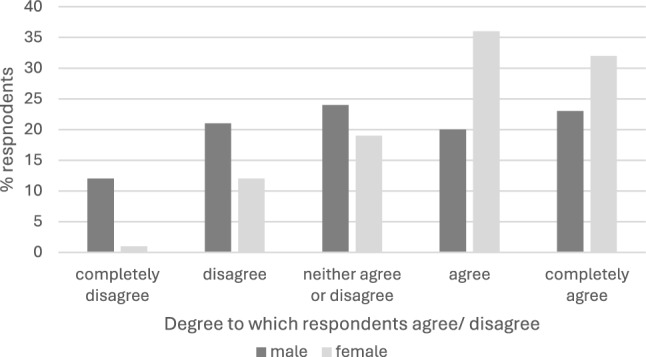
Percentage of respondents who agree/disagree with the following statement: “I feel comfortable asking female patients about most aspects of their sex life when indicated” by respondent sex

**Fig. 2:**
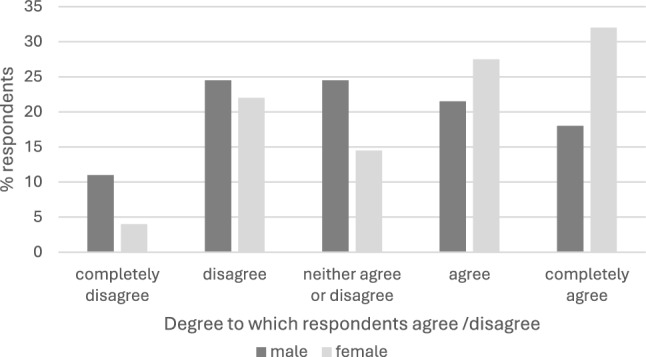
Percentage of respondents who agree/disagree with the following statement: “I feel comfortable asking female patients about anal sex when indicated” by respondent sex

Respondents ease of enquiry about sex generally and AI are shown in Table 5 and Figs. 1 and 2. Potential barriers to discussing AI with women in the clinical scenario are presented in Table 6. Discomfort with general sexual and AI enquiry was seen. Barriers relate to perceptions and sensitivities. Male clinicians experienced greater barriers with the difference most striking amongst consultants; 59% of male CCS said fear the patient would think them creepy was a barrier c.f. 10% of female colleagues.

**Table 5:** Ease of enquiry about AI in the clinical scenario

“To what extent do you agree with the following statements?”						
	All respondents (%) *n* = 167		Colorectal consultants (%) *n* = 75		Pelvic floor specialists (%) *n* = 14	
I feel comfortable asking female patients about most aspects of their sex life when indicated	Completely agree	44 (26)	Completely agree	21 (28)	Completely agree	5 (36)
	Agree	45 (27)	Agree	24 (32)	Agree	5 (36)
	Neither agree or disagree	36 (22)	Neither agree or disagree	12 (16)	Neither agree or disagree	1 (7)
	Disagree	29 (17)	Disagree	24 (32)	Disagree	2 (14)
	Completely disagree	13 (8)	Completely disagree	6 (8)	Completely disagree	1 (7)
I feel comfortable asking female patients about anal sex when indicated	Completely agree	40 (24)	Completely agree	21 (28)	Completely agree	6 (43)
	Agree	40 (24)	Agree	24 (32)	Agree	4 (29)
	Neither agree or disagree	34 (20)	Neither agree or disagree	9 (12)	Neither agree or disagree	1 (7)
	Disagree	39 (23)	Disagree	16 (21)	Disagree	2 (14)
	Completely disagree	14 (8)	Completely disagree	15 (20)	Completely disagree	1 (7))

**Table 6:** Potential barriers to discussing AI with women in the clinical scenario

To what degree do any of the following act as a barrier to taking a history of anal sex from female patients?						
	All respondents (%) *n* = 167		Colorectal consultants (%) *n* = 75		Pelvic floor specialists (%) *n* = 14	
Lack of time in the clinic	Not a barrier	66 (40)	Not a barrier	34 (45)	Not a barrier	3 (21)
	2	22 (13)	2	7 (9)	2	1 (7)
	3	30 (18)	3	13 (17)	3	4 (29)
	4	27 (16)	4	12 (16)	4	3 (21)
	Significant barrier	22 (13)	Significant barrier	9 (12)	Significant barrier	3 (21)
Concern the patient will think I’m creepy	Not a barrier	58 (35)	Not a barrier	29 (39)	Not a barrier	8 (57)
	2	24 (14)	2	8 (11)	2	0
	3	22 (13)	3	8 (11)	3	0
	4	34 (20)	4	16 (21)	4	4 (29)
	Significant barrier	29 (17)	Significant barrier	14 (19)	Significant barrier	2 (14)
Fear of being perceived judgemental	Not a barrier	40 (24)	Not a barrier	21 (28)	Not a barrier	8 (57)
	2	20 (12)	2	11(15)	2	0
	3	28 (17)	3	8 (11)	3	1 (7)
	4	52 (31)	4	24 (32)	4	3 (21)
	Significant barrier	27 (16)	Significant barrier	11 (15)	Significant barrier	2 (14)
Embarrassment	Not a barrier	59 (35)	Not a barrier	34 (45)	Not a barrier	9 (64)
	2	37 (22)	2	21 (28)	2	1 (7)
	3	28 (17)	3	8 (11)	3	2 (14)
	4	29 (17)	4	10 (13)	4	2 (14)
	Significant barrier	14 (8)	Significant barrier	2 (3)	Significant barrier	0
Lack of experience asking about sex	Not a barrier	80 (48)	Not a barrier	49 (65)	Not a barrier	8 (57)
	2	24 (14)	2	9 (12)	2	2 (14)
	3	32 (19)	3	7 (9)	3	1 (7)
	4	19 (11)	4	6 (8)	4	1 (7)
	Significant barrier	12 (7)	Significant barrier	4 (5)	Significant barrier	2 (14)
Lack of understanding as to when it is relevant	Not a barrier	71 (42)	Not a barrier	45 (60)	Not a barrier	11 (79)
	2	26 (16)	2	10 (13)	2	1 (7)
	3	37 (22)	3	11 (15)	3	1 (7)
	4	22 (13)	4	6 (8)	4	0
	Significant barrier	11 (7)	Significant barrier	3 (4)	Significant barrier	1 (7)
Shortage of chaperones	Not a barrier	74 (44)	Not a barrier	40 (53)	Not a barrier	5 (36)
	2	27 (16)	2	13 (17)	2	4 (29)
	3	30 (18)	3	12 (16)	3	3 (21)
	4	11 (7)	4	4 (5)	4	0
	Significant barrier	25 (15)	Significant barrier	6 (8)	Significant barrier	2 (14)
Fear of making the patient uncomfortable	Not a barrier	18 (11)	Not a barrier	10 (13)	Not a barrier	2 (14)
	2	26 (16)	2	14 (19)	2	5 (36)
	3	27 (16)	3	15 (20)	3	1 (7)
	4	57 (34)	4	26 (35)	4	4 (29)
	Significant barrier	39 (23)	Significant barrier	10 (13)	Significant barrier	2 (14)
My personal beliefs	Not a barrier	148 (89)	Not a barrier	71 (95)	Not a barrier	13 (93)
	2	4 (2)	2	3 (4)	2	0
	3	9 (5)	3	1 (1)	3	1 (7)
	4	3 (2)	4	1 (1)	4	0
	Significant barrier	2 (1)	Significant barrier	0	Significant barrier	0

Respondents indicated little confidence in commonly cited harm reduction techniques (Table 7); 95% of CCS and 100% of PFS said healthcare worker training and public health awareness about possible health consequences are needed (Table 8).

**Table 7:** Opinion about protective mechanisms “To what extent do you agree with the following statements?"

	All respondents (%) *n* = 167		Colorectal consultants (%) *n* = 75		Pelvic floor specialists (%) *n* = 14	
It is possible to adequately protect the anal sphincter by using lubrication during anal sex	Completely agree	15 (9)	Completely agree	6 (8)	Completely agree	1 (7)
	Agree	21 (13)	Agree	7 (9)	Agree	1 (7)
	Neither agree or disagree	37 (22)	Neither agree or disagree	14 (19)	Neither agree or disagree	1 (7)
	Disagree	28 (17)	Disagree	15 (20)	Disagree	5 (36)
	Completely disagree	17 (10)	Completely disagree	12 (16)	Completely disagree	4 (28)
	Don’t know	49 (29)	Don’t know	21 (28)	Don’t know	2 (14)
It is possible to adequately protect the anal sphincter by relaxation techniques during anal sex	Completely agree	14 (8)	Completely agree	3 (4)	Completely agree	0
	Agree	21(13)	Agree	5 (7)	Agree	2 (14)
	Neither agree or disagree	38 (23)	Neither agree or disagree	19 (25)	Neither agree or disagree	2 (14)
	Disagree	23 (14)	Disagree	14 (19)	Disagree	3 (21)
	Completely disagree	17 (10)	Completely disagree	13 (17)	Completely disagree	5 (36)
	Don’t know	54 (32)	Don’t know	21 (28)	Don’t know	2 (14)

**Table 8:** Opinions on education and training

	All respondents (%) *n* = 167	Colorectal consultants (%) *n* = 75	Pelvic floor specialists (%) *n* = 14
Do you feel there is a need for increased training amongst healthcare workers about anal sex in women?			
Yes	159 (95)	71 (95)	14 (100)
No	No 8 (5)	4 (5)	0
Do you feel there is a need for increased public health awareness about the possible health consequences of anal sex by women?			
Yes	162 (97)	71 (95)	14 (100)
No	5 (3)	4 (5)	0

## Discussion

Online questionnaires are quick, easy and cheap, but lack of time, interest or discomfort with subject matter may inhibit completion. As some ASGBI members do not see relevant patients, we anticipated fewer responses from them. Individuals who are members of both societies received the survey twice, so it is not possible to describe our response rate for each organisation. However, 1212 individuals are currently listed as members of the ACPGBI [15], and a 2023 Royal College of Surgeons (RCSE) census found 928 colorectal surgeons in England and Wales [16], so our response rate appears low.

Only 41% of individuals who visited our survey online completed it. Anal sex has been a taboo subject [7] and it is possible this affected response rates. A recent survey about elderly patients distributed via the ACPGBI achieved a 40% response rate [17]. We chose to use the term anal sex and did not go into graphic detail about the various types of anal penetration in order to minimise the risk of our survey putting off or even offending some potential participants. We chose multiple choice questions, as they are quick and easy for respondents, work well on a mobile phone and provide standardised answers allowing data analysis. The responses we received, particularly regarding clinician-related barriers to enquiry about AI, can now direct qualitative research on smaller groups of clinicians.

Only 15% of colorectal surgeons, in the RCSE survey were female compared to 41% of our respondents [16]. Our survey, therefore, disproportionately attracted responses from female clinicians. Perhaps women have stronger views about the subject; however, this could simply reflect different characteristics between male and female surgeons [18] and their willingness to complete surveys.

CCS and PFS are aware women have less robust ani than men, with shorter sphincters and lower sphincter pressures [19, 20]. They also suffer threats to continence from pregnancy and parturition [21]. Where direct observation is limited, clinicians use comparable situations to guide them. CCS know the effect anal stretching has on continence [22] and that IAS damage is associated with surgical stapling devices [23, 24], so they can make inferences as to the effect insertion of larger objects under less controlled conditions may have.

It is therefore unsurprising most CCS and PFS agreed female AI can injure the IAS and cause FI in women. Only 4% of CCS disagreed female AI can cause IAS injury and only 2% FI. No PFS disagreed with either statement. This suggests those colorectal specialists who responded to our survey think female AI can lead to significant pathology, including future FI. Higher rates of FI amongst women engaging in AI (9.9% vs. 7.4%, *p* = 0.05) c.f. those not engaging has been reported by adults in a US cross-sectional study [25].

Given the anatomical and reproductive differences, it is reasonable to hypothesise AI poses a greater threat to female continence c.f. men. Forty-seven per cent of CCS and 57% of PFS agree with this. However, there was uncertainty, with 17% of CCS disagreeing and a quarter responding “don’t know”. This may reflect a paucity of information about the effects of ano-receptive sex across all genders.

Anal pain, bleeding and fissures [26, 27] are described consequences of AI, and our respondents were aware of this. However, there was dissonance between knowledge and practice. Rates of enquiry about AI were lower than predicted given opinions about causation. Ninety-one per cent of CCS agreed female AI can cause anal fissures, yet only 44% felt it was important to ask about it and only 25% would usually ask a 19-year-old woman with a fissure and one in three respondents said they would never ask.

ACPGBI guidelines highlight the prevalence of AI stating, “It is important to ascertain sexual history during the assessment of patients with anal fissure”. The guideline recommends conservative management of AI-associated fissures [28]. Suboptimal history taking thereby exposes women to futile or inappropriate treatment and denies them the opportunity to consider lifestyle change.

The UK National Health Service (NHS) has policies and procedures in place to protect vulnerable patients, defined as those who cannot care for themselves or protect themselves from harm. All NHS clinical staff are taught to specifically recognise and care for these patients, and our survey found enquiry into AI increased with patient vulnerability, with 57% of both CCS and PFS usually asking. In Belgium, ano-receptive intercourse was implicated in 13% of chronic fissures and sexual abuse found in 19% [29]. Higher rates of enquiry with vulnerable patients suggest sensitivity to this. However, 11% of CSS would still never ask and half our respondents would usually not ask.

Some respondents indicated they ask some patients but not others, i.e. making judgements. This is unlikely to be a robust method for differentiating those for whom the question is relevant. The reasons teenagers and young women cite for AI do not generally show a high level of agency. Motives are often pleasing the male partner, maintaining relationships and meeting societal norms [10–13]. These individuals would not be classed as “vulnerable patients” [30]. However, society does place them in a weak position with coercion to participate in AI being sufficiently common that it is normalised in UK teen culture [31]. Failure of clinicians to routinely ask about AI may represent missed opportunities to support those who are not necessarily acting in their own interests.

We saw a similar pattern with incontinence: 80% of CCS and 85% of PFS agreed female AI can cause IAS damage, but only 56% and 64% respectively felt it important to ask incontinent nulliparous women about it, and even less say they actually ask.

Clinicians perhaps expect patients to proffer information about AI and ask the clinician about its relevance. Qualitative studies indicate this is unlikely to be safe practice. Social stigma limits women’s ability to discuss AI. Men are praised for AI conquests and women are shamed [32]. This double standard may inhibit female patients’ ability to “confess” to AI to a clinician.

Time constraints were cited as a barrier to enquiry about AI. The question and reply take moments, so perhaps this indicates fear of “opening a can of worms”. Fear of being thought creepy was a particular concern for male clinicians, perhaps explaining the difference between awareness and practice. The higher rates of enquiry by male clinicians in the intractable fissure scenario may indicate acceptance that now the question is unavoidable, despite the fear it engenders.

Fear of making patients uncomfortable or appearing judgemental are obstacles for clinicians of both sexes. Conversely, embarrassment and personal beliefs were not. The latter is borne out by logistic regression analysis, which showed no evidence that beliefs, age or ethnicity affected willingness to discuss AI with female patients.

Ileo-anal pouch surgery for inflammatory bowel disease is performed by subspecialists. A third of CCS said they never counsel women for the procedure. Of the remainder, a third said they always discuss AI, but one in five said they never discuss it. Inexplicably, 50% sometimes discuss it, indicating knowledge of the issues but selectivity as to which patients are made aware, or perhaps only discussing AI if patients raise it. Patient-led research highlights the paucity of sex information around colorectal surgery generally [33], and the sudden increase in AI by women may mean guidelines and patient information systems are lagging behind societal norms.

NatSALs are some of the largest and most detailed studies of sexual behaviour worldwide. They have been interviewing UK residents since 1990 [4]. The first three surveys investigated experience of AI. In 1990–1991, few women reported AI. Data from 1999 to 2001 showed 24% of women under 25 years and 28.5% of women under 35 years had experienced AI, increasing to 29% and 39% respectively during 2010–2012. We saw a range in respondents' prevalence estimates, with the median in line with the 2012 figures. Current data are not available, as Natsal 4 concentrated on sex during the COVID pandemic. However, if the increase in female AI continued along a similar trajectory, by 2023 more than half of women under < 35 years will have experienced it.

Overwhelmingly, our respondents want more information about female AI for both healthcare workers and the public. This is reflected in other sections of our survey by “don’t know” responses. A plethora of non-medical and pseudo-medical websites currently fill the health information void. Rather than helping women make informed decisions, they may give unfounded advice and increase societal pressure to participate.

Websites, marketers and some medical professionals advocate women lubricate and practice relaxation to mitigate the adverse consequences of AI. Our respondents were sceptical about their value. Given the autonomic innervation of the IAS and the fact that it gets damaged by lubricated surgical staplers under general anaesthetic [23, 24], this scepticism is unsurprising.

This is the first study to our knowledge to investigate the opinions of colorectal specialists about female AI. Expert opinion suggests high levels of concern and the need for objective measurement of anal pathology and sphincter pressure in women having AI. Until such definitive studies are available, specialists’ opinions are useful to guide other clinicians and health educators. This is particularly important as there is so much unsubstantiated information available elsewhere. Raising awareness aids conversations.

## Data Availability

No datasets were generated or analysed during the current study.
